# Comparison of Ketamine and Tramadol in Management of Acute Pain; a Systematic Review

**Published:** 2020-08-23

**Authors:** Bahman Naghipour, Mahboub Pouraghaei, Ali Tabatabaey, Allahveirdy Arjmand, Gholamreza Faridaalaee

**Affiliations:** 1; 2; 3; 4; 5

**Keywords:** Ketamine, Tramadol, pain management, emergency treatment, acute pain

## Abstract

**Introduction:**

Management of pain is an important part of care in the emergency department (ED). Tramadol and Ketamine have both been introduced as alternatives to opioids in the ED and post-operative setting. In this study, we conducted a systematic review of available literature to compare the analgesic efficacy, and side effect profile of these two medications in management of severe acute pain.

**Methods:**

This is a systematic review based on the PRISMA protocol. In this study, peer-reviewed papers published by March 3, 2020, which compared analgesic effects of tramadol and ketamine in management of acute pain were included.

**Result:**

The initial search of online databases identified 2826 non-duplicate records. Finally, three papers available in full text were analyzed for study quality. The results show that ketamine has consistently been shown to be superior to tramadol for pain control and causes fewer significant side effects.

**Conclusion:**

Results of this review show that low-dose ketamine is more effective than tramadol in pain control, while causing fewer side effects.

## Introduction

Pain is one of the most common patient complaints in the emergency department (ED), and management of pain is an important part of care in the ED (1). Multicenter studies have reported that up to 78% of patients admitted to ED complain of acute pain (1). Pain control has been considered a human right (2) and in 2011, analgesics were administered in 97 million ED visits (3). Many medications are used as analgesics for acute pain; examples include opioids, non-steroidal anti-inflammatory drugs, acetaminophen, ketamine, and duloxetine (4-8). Educational campaigns in the 1990s focused on systematic assessment and treatment of pain (9). A perfect analgesic is one with quick onset, no side effects, and an extended effect. Since, such a medication has yet to be discovered strategies such as “analgesic pyramid”, “balanced analgesia” and “Channels-Enzymes-Receptors Targeted Analgesia” have emerged (10). In the analgesic pyramid, medication is chosen based on severity of pain. Yet, for severe pain, treatment options are limited and opioids are the most common analgesic used (9), despite their unfavorable adverse effect profile, including respiratory depression, dependence, and risk of overdose. In Canada, one in every 550 people who are started on opioids die (11), and death from an opioid overdose is an important cause of death in the 18 to 35 age group (11, 12). Therefore, clinicians are looking at safer alternatives for the management of severe pain in the postoperative and ED settings. 

Tramadol is considered an atypical opioid with multiple effects on various receptors (13, 14). It has shown efficacy in reducing different types of pain and is less likely to cause dependence than opioids. Its adverse effect profile is different from opioids and is less likely to cause respiratory depression but more likely to induce seizures (7, 13, 15-17).

In recent years, ketamine has gained popularity as a sedative and analgesic in the ED. Ketamine is a phencyclidine derivative, which is a dissociative sedative and an amnestic in addition to having analgesic properties (6). It has also been found to have fast acting antidepressant effects (18). It can easily be administered through oral, intranasal, rectal, intramuscular, or intravenous routes. It has rapid onset and a wide window of effects without the risk of respiratory depression, which makes it an attractive choice for management of acute pain. Yet fear of other side effects, such as increased intracranial pressure, increased cardiovascular load, and emergence reactions, has limited its use (19-22).

Ketamine and tramadol both have advantages and disadvantages, so in this study we decided to systematically review available literature to compare the analgesic efficacy, and side effect profile of these two medications in management of severe pain. 

## Methods

***Study setting***

This is a systematic review based on the PRISMA protocol (23). The study PICO is: Population of patients with severe acute pain in the ED or postoperative setting, Intervention is Ketamine, intravenously, compared with tramadol, and the Outcome is control of acute pain. Secondary outcome is the prevalence of side effects. 

***Eligibility criteria***

In this study, peer-reviewed papers published by March 3, 2020, which compared analgesic effects of tramadol and ketamine for in management of acute pain were included. Intravenous administration of ketamine and tramadol was an inclusion criteria and other routes of administration, such as nasal or epidural routes, were excluded from the study. For papers that were only available as abstracts, multiple attempts were made to contact the authors using available means (emails, social media, researchgate, etc.). Unfortunately, we were not able to secure full text versions for abstracts presented in seminars. 

***Search Strategy***

Relevant search items and keywords for the study question were selected from MeSH and Emtree terms after consulting an Emergency Medicine specialist and an Anesthesiologist. A literature search was conducted via electronic resources including Medline, Web of Science, Embase, and Central Cochrane Library up to the March 3, 2020. References of found papers were also searched for relevant studies. The search strategy in Medline followed the pattern described in table 1.

**Table 1:** Medline search strategy

Database	**Search terms**
MEDLINE (PubMed)	Ketamine OR Ketalar OR Ketaset OR Ketanest OR Calipsol OR Kalipsol OR Calypsol OR Ketamine Hydrochloride OR Esketamine Tramadol OR Tramundin OR Biodalgic OR Jutadol OR MTW-Tramadol OR MTW Tramadol OR MTWTramadol OR Nobligan OR Prontofort OR Zytram OR Takadol OR Theradol OR Tiral OR Tramadol Lindo OR Topalgic OR Tradol OR Tradol-Puren OR Tradol Puren OR TradolPuren OR Tradonal OR Tralgiol OR Trama AbZ OR Trama KD OR Trama-Dorsch OR Trama Dorsch OR TramaDorsch OR Biokanol OR Tramabeta OR Tramadin OR Tramadol-ratiopharm OR Tramadolratiopharm OR Tramadol Ratiopharm OR Tramadoc OR Tramadol PB OR Tramadol acis OR Tramadol AL OR Tramadol Basics OR Tramadol Bayvit OR Tramadol Bexal OR Tramadol 1A OR Ranitidin 1A Pharma OR Trama 1A Pharma OR Tramadol Cinfa OR Tramadol Edigen OR Tramadol Hydrochloride OR Trasedal OR Ultram OR Tramadol Heumann OR Xymel 50 OR Zamudol OR Zumalgic OR Zydol OR Tramadol Kern OR Tramadol Lichtenstein OR Tramadol Mabo OR Tramadol Normon OR Tramadol Stada OR Tramadol-Dolgit OR Tramadol Dolgit OR TramadolDolgit OR Tramadol-Hameln OR Tramadol Hameln OR TramadolHameln OR Tramadolor OR Tramadura OR Tramagetic OR Tramagit OR Tramake OR Tramal OR Tramex OR Adolonta OR Contramal OR Amadol OR Tramadol Asta Medica 1 &2

***Study selection, data collection, and outcome measurement***

Initially, all the studies found, which evaluated effects of ketamine and tramadol in management of acute pain, were included. Abstracts of all papers were reviewed by two members of the research team and papers were further screened based on inclusion and exclusion criteria described above. Relevant papers were then reviewed in full text and those meeting the criteria were included in the study. Their findings were then summarized and evaluated using standardized checklists and study quality was assessed by two members of the research team, independently. Any discrepancy between the reviews was resolved either through discussion or a verdict by a third researcher. In this systematic review, the primary outcome was analgesic efficacy of tramadol and ketamine, described through decrease in pain score or necessity of a second analgesic. Secondary outcome was prevalence of adverse effects among patients receiving tramadol and ketamine. 

***Statistical analysis***

Descriptive analysis was performed on data. All included studies were summarized and categorized based on predefined variables. 

## Results

***Study selection and study characteristics***

**Figure 1:**
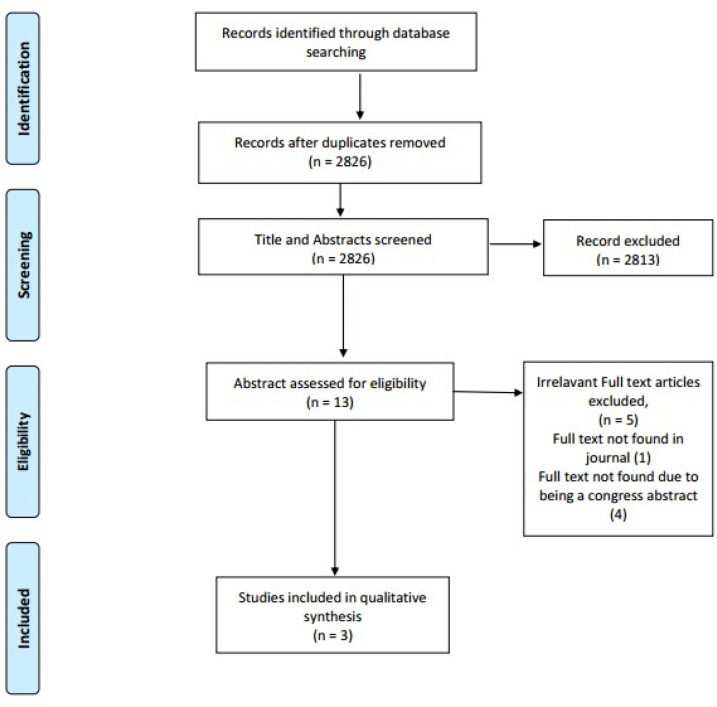
PRISMA diagram of systematic review

The initial search of online databases identified 2826 non-duplicate records. After screening of abstracts, 2813 studies were eliminated. Thirteen papers were assessed in full text. Five were found to be ineligible for the study. Four studies were presentation abstracts and attempts were made to contact authors in order to obtain them in full text without success (24-27). Two of the presentations consisted of similar findings (26, 27). One paper was only available in abstract form and the author did not respond to researchers (28). Therefore, only three papers, available in full text, were analyzed for study quality (29-31). Due to the paucity of eligible studies, the authors decided to include findings from the presentation abstracts with relevant findings in this systematic review. Figure 1 summarizes the selection process. Specifications and characteristics of included studies are reported in table 2. Overall, 257 patients were evaluated for analgesic effects of ketamine and tramadol in acute pain. 

***Quality control of study and risk of bias***

The quality of included studies was evaluated, and results are summarized in Table 3.

**Table 2:** Characteristics of included studies

Author	Ketamine group				Tramadol group			
	N	Age	Male	Dose	N	Age	Male	Dose
Khajavi 2016	40	46.93	29	0.5 mg/kg	40	42.17	25	0.7 mg/kg
Burimsittichai 2016	70	47	14	0.5 mg/kg	67	44	12	1.5 mg/kg
Yu C 2005	20	44.67	14	0.5 mg/kg	20	47.33	9	0.3 mg/kg
Kilinc Y 2018	16	7-21	10	Ketamine (0.25 mg/ kg/ dose)	31	6-21	16	tramadol (0.1-0.4 mg /kg/ h)
Zghidi 2011	20	adult	-	0.2mg/kg, + 2µ/kg/min	20	adult	-	100 mg + 0.5 mg/kg and 0.1mg/kg/h

**Table 3:** Quality control of included studies

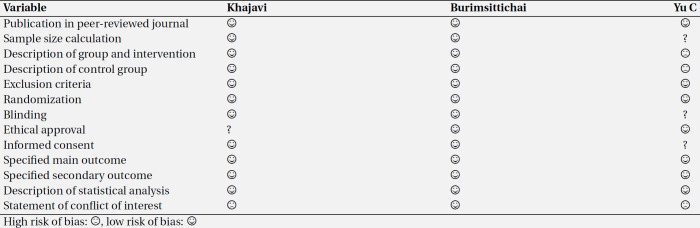

***Analgesic effect***

Systematic review of results shows that analgesic effects of low-dose ketamine (0.25-0.5 mg/kg) are significantly larger/stronger than tramadol (0.3-1.5 mg/kg).

Khajavi et al. looked at 80 patients with acute pain following renal surgery in a double blind randomized controlled study (29). One group received intravenous paracetamol plus ketamine and the other received intravenous paracetamol plus tramadol. They found that patients who received ketamine had significantly lower pain scores compared to those who received tramadol. In the study by Burimsittichai et al. 207 patients with pain secondary to foley catheters in the postoperative setting were randomly allocated to a group in a double blind randomized clinical trial (RCT) to receive either ketamine 0.5mg/kg or tramadol 1.5mg/kg, or placebo (30). Analgesic effects were compared 6 and 24 hours after laparoscopic surgery. The authors reached the conclusion that use of either medication significantly reduces pain, compared to placebo, but did not compare these groups to one another. Although the group receiving ketamine appeared to have lower pain scores, the statistical significance of this observation was not discussed by the authors. Yu C. and colleagues studied patients suffering from postoperative hyperalgesia and also found that those receiving intravenous ketamine had significantly better pain control compared to those receiving tramadol (31). 

Kilinc and colleagues in their study looked at patients with sickle cell anemia, aged 6 years and over, suffering from acute pain episodes (25). One group received ketamine and the other received tramadol. Results showed that ketamine was more effective that tramadol in controlling pain. Alp reported using either ketamine 0.5mg/kg or tramadol 1mg/kg for pain control in 100 women undergoing uterine dilatation and curettage in Turkey (24). In the results presented at the 35^th^ Annual European Society of regional Anaesthesia and Pain Therapy, they reported significantly better pain control in the group receiving ketamine. At the 30^th^ Annual European Society of regional Anaesthesia, a study from Tunisia, presented by Zghidi, reported the findings of comparison of ketamine and tramadol in controlling post laparotomy pain in 40 patients and revealed that ketamine was a more effective analgesic (26). These results are similar to another study by Djaziri, presented at 32^nd^ Annual European Society of regional Anaesthesia and Pain Therapy (27).

***Need for rescue analgesic***

Khajavi et al. used morphine as their rescue analgesic and showed that those receiving ketamine had significantly lower rescue morphine injections during the first 6 hours (0.47 ± 0.94 mg versus 2.50 ± 1.35 mg, P = 0.001) (29). Burimsittichai and colleagues, on the other hand, did not see any significant difference between the groups regarding rescue morphine therapy (30). 

***Side effect profile***

Evaluation of included studies shows that ketamine has a safer side effect profile compared to tramadol.

In the study by Khajavi and colleagues, patients’ agitation was measured, using the Ramsey Scale Score, 10 and 20 minutes after the injection of analgesics (29). The group receiving ketamine had significantly lower agitation compared to the group receiving tramadol. Also, the prevalence of side effects was significantly lower in the ketamine group (20% for ketamine and 53% for tramadol). Patients receiving tramadol experienced higher rates of nausea, vomiting, and hallucinations but the difference between the two groups was not statistically significant. 

In the study by Burimsittichai et al. rates of nausea and vomiting were higher in the ketamine group, but the difference was not found to be significant compared to the either the tramadol group or the placebo group (30). The groups were found to have comparable rates of other side effects. In the study by Yu C. the two groups were not significantly different regarding incidence of side effects (31). In a study by Elkassem, effects of ketamine and tramadol were compared in patients undergoing cruciate ligament reconstruction surgery and it was found that tramadol had higher rates of side effects compared to ketamine (28). In the study by Alp, ketamine was reported to have higher rates of side effects, which included increased heart rate and blood pressure (24).

## Discussion

 This systematic review aimed to explore existing literature to compare the analgesic effects of ketamine and tramadol in treating acute pain and compare their side effect profiles. Results of this review show that low-dose ketamine is more effective than tramadol in pain control, while causing fewer side effects. Three full text papers were included in this study alongside four studies only reported as abstract presentations in scientific gatherings. The authors concluded that these results demonstrate that use of ketamine for acute pain control is advisable. 

Several systematic reviews have looked at analgesic effects of ketamine in the past. In 2018 Karlow et al. looked at literature comparing low-dose ketamine to morphine for treatment of acute pain in the ED (32). This review included three studies and reached the conclusion that low-dose ketamine is equally effective as morphine in pain control, while having fewer significant side effects. They suggested using ketamine instead of morphine for treatment of severe pain in the ED (32). Ghate and colleagues also looked at the effects of low-dose ketamine (0.15-0.3mg/kg) for pain control in the ED (33). They included 6 RCTs and two observational studies. The authors concluded that ketamine is as effective as morphine in controlling pain and patients reported similar satisfaction rates. Side effects reported for using ketamine included dysphoria, hallucinations, agitation, and confusion (33). A systematic review by Lee and colleagues in 2016 included 6 studies and looked at the effectiveness of ketamine in patients with moderate to severe pain in the ED (34). They concluded that ketamine had comparable effects with morphine but it was found to be inferior to fentanyl in two studies. One of the included studies had found that ketamine was not superior to placebo at 0.15mg/kg and only showed analgesic effects at 0.3 mg/kg. The authors found that ketamine caused more neurologic (dizziness, headache, light-headedness, nystagmus, visual disturbance, drowsiness, numbness, or increased skeletal tone) and psychological (hallucination, dysphoria or confusion, agitation, disorientation, or mood change) side effects compared to morphine but had fewer cardiac side effects (major: hypoxia and hypotension; minor: tachycardia and hypertension) (34). 

Other studies have also looked at ketamine in different settings. Yousefifard et al. recently looked at the effects of ketamine in the prehospital setting and found that ketamine is less effective than morphine and fentanyl, but it also has fewer side effects (35). Others have looked at ketamine in the perioperative setting. Wang et al. looked at 20 clinical trials in cesarian sections (36), while Riddell et al. looked at orthopedic surgeries (37), and García-Henares JF. et al. looked at general surgery patients (38). All of these reviews found that ketamine is effective in reducing postoperative pain and decreasing the need for opioids without causing an increase in significant side effects. 

Our results show that ketamine has consistently been shown to be superior to tramadol in pain control, also causing fewer significant side effects. Therefore, we suggest the use of ketamine instead of tramadol as an opioid-spearing analgesic in treatment of acute pain.

## Limitation

We only found three articles that compared the analgesic effects of Ketamine and tramadol, so we did not perform a meta-analysis. Risk of bias for all three articles is shown in table 3. All three articles have low risk of bias regarding main and secondary outcomes. 

## Conclusion:

The results of this review show that low-dose ketamine is more effective than tramadol in pain control, while causing fewer side effects.
